# Identification and validation of a ferroptosis-related signature for prediction of the prognosis and tumor microenvironment in patients with chromophobe renal cell carcinoma

**DOI:** 10.1186/s12885-023-11589-5

**Published:** 2023-11-08

**Authors:** Shuai Liu, Yu Yao, Mingyu Hou, Jingchang Mei, Lijiang Sun, Guiming Zhang

**Affiliations:** 1 Department of Urology, The Affiliated Hospital of Qingdao University, No. 16, Jiangsu Rd, 266003 Qingdao, P.R. China; 2 Department of Pathology, The Affiliated Hospital of Qingdao University, 266003 Qingdao, P.R. China

**Keywords:** Chromophobe renal cell carcinoma, Ferroptosis, Molecular subtype, Prognostic signature, Immune microenvironment

## Abstract

**Background:**

Ferroptosis is a novel form of regulated cell death that is different from other forms, which has an important role in tumor growth inhibition. The purpose of this study was to construct and validate a prognostic signature related to ferroptosis in chromophobe renal cell carcinoma (ChRCC) and to explore its role in immune cell infiltration and systemic therapy.

**Methods:**

The gene expression profiles of ChRCC patients obtained from The Cancer Genome Atlas (TCGA) database were used to identify differentially expressed prognostic ferroptosis-related genes (FRGs) by univariate Cox proportional hazards analyses. Ferroptosis molecular subtypes were obtained by consensus clustering analysis. The FRG-based signature in the training set was established by least absolute shrinkage and selection operator analysis and verified in the testing set. The association between molecular subtypes and the prognostic signature and immune microenvironment was explored to predict responses to immunotherapy. Immunohistochemistry was used to verify expression of the FRG-based signature externally.

**Results:**

ChRCC patients were divided into two FRG subtypes. Two FRGs (TFRC and SLC7A11) were identified to construct the prognostic signature. The high-risk group and cluster 2 had worse overall survival than the low-risk group and cluster 1, respectively. The low-risk group and cluster 1 had higher levels of immune cell infiltration and expression of MHC and immune checkpoint molecules than the high-risk group and cluster 2. The risk score was a predictor of overall survival and had a good predictive ability, which was verified in the testing set and evaluated by ROC and calibration curves. The high-risk group had a higher tumor mutation burden. The different sensitivities of targeted drugs in patients with different risks were evaluated. External immunohistochemical analysis showed that TFRC and SLC7A11 were highly expressed in tumor tissues compared with para-cancer normal tissues, and the expression level was significantly associated with a more advanced stage and worse cancer-specific survival.

**Conclusions:**

An FRG signature was identified and validated to predict the clinicopathological features and prognosis of ChRCC. A significant association between the signature and immune cell infiltration, immune checkpoint expression, and drug response is helpful to guide comprehensive treatment of ChRCC.

**Supplementary Information:**

The online version contains supplementary material available at 10.1186/s12885-023-11589-5.

## Background

The incidence of renal cell carcinoma (RCC) is increasing yearly, causing approximately 175,098 deaths each year [1, 2]. Chromophobe renal cell carcinoma (ChRCC) is a subtype of RCC, accounting for approximately 5% of cases [3]. ChRCC has a good prognosis with a 10-year cancer-specific survival rate of approximately 89%, but 8.6–12.7% of patients have recurrence and metastasis [4, 5]. Tumor stage, lymph node metastasis and distant metastasis were commonly used to predict prognosis and guide treatment. There has been no suitable pathological grading system of ChRCC for clinical practice [6]. However, only TNM staging is not sufficient to predict the ChRCC patient’s prognosis and develop a treatment plan [7, 8]. Early identification of these patients with poor prognoses and a high risk of metastases is a hot topic for clinical research, in which genetic biomarkers play an important role.

Ferroptosis is a novel type of cell death that is distinct from apoptosis, autophagy, necroptosis, and pyroptosis. It is dependent on iron and reactive oxygen species to generate membrane lipid peroxidation and oxidative stress that disrupts permeability of the plasma membrane [9]. Ferroptosis markers associated with prognosis has been found in numerous tumors such as lung cancer, breast cancer, gastric cancer and clear cell renal cell carcinoma (ccRCC), and these studies constructed ferroptosis related prognostic signature [10–13]. Ferroptosis-related genes (FRGs) were regulated by the tumor suppressor TP53 to inhibit tumor growth [14]. Therefore, many ferroptosis inducers have been developed to treat malignancy [15]. Gao et al. found that ferroptosis related signature was closely related to immunotherapy and predicted the effectiveness of immunotherapy for ccRCC [16]. Moreover, ferroptosis related pathway (TAZ/WNT10B) was found to be a tumor immune-related pathway and a potential target for immune checkpoint inhibitor therapy of ccRCC [16]. In the tumor microenvironment, cancer cells and immune cells release lots of chemokines and cytokines to regulate the development of tumor. It was previously reported that ferroptosis inhibited the activity of tumor-infiltrating immune cells (such as CD8^+^ cells, natural killer cells and dendritic cells) and caused functional impairment [17]. On the other hand, some immunosuppressive immune cells, including M2 tumor-associated macrophages and T regulatory cells, also need FRGs, such as Glutathione peroxidase 4 (GPX4), to suppress ferroptosis and maintain cell activation [17]. However, the association of FRGs and prognosis and immunotherapy of ChRCC has not been investigated so far.

The aim of our study was to investigate an FRG signature and molecular subtype related to ChRCC prognosis and validate them in our medical center. We also analyzed the predictive role of the signature and cluster in immune cell infiltration, tumor mutation burden, immune checkpoint expression, and sensitivity to potential drugs.

## Materials and methods

### Data collection

Gene expression profiles, corresponding clinical information, and somatic mutations of ChRCC patients were downloaded from The Cancer Genome Atlas (TCGA) official website (https://portal.gdc.cancer.gov/). Sixty FRGs were retrieved from previous studies [18–21] and are shown in Table S1.

### Gene mutation summary and identification of differentially expressed FRGs

A waterfall plot was used to show the frequency and type of mutation in ChRCC samples. Differentially expressed FRGs between ChRCC and normal tissues were identified using the “limma” R package. The cutoff value was set to false discovery rate < 0.05 and |log2 fold change|>1. The heatmap and volcano plot were used to demonstrate the expression difference of genes between tumor and normal samples. The STRING (Search Tool for the Retrieval of Interacting Genes) (Version 11.5) website was used to generate protein–protein interaction (PPI) networks of differentially expressed FRGs.

### Cluster analysis

The “survival” R package was used to perform univariate Cox proportional hazards regression models and obtain survival-related FRGs. Cluster analysis (K-means) based on Euclidean distance used “ConsensusClusterPlus” R packet to identify molecular subtypes related to ferroptosis. The number of repetitions is 50 and the proportion of the subsample is 0.8. The Kaplan–Meier method was used to compare overall survival between clusters.

### Construction and validation of an FRG-based prognostic risk signature

All patients were randomly divided into the training and testing sets by the “caret” R package. The grouping was performed according to a 1:1 ratio of the training set and testing set. The best FRG-based signature to predict ChRCC prognosis was constructed by least absolute shrinkage and selection operator (LASSO) Cox regression analysis using the “glmnet” R package on the basis of the best lambda (λ) in the training set [22, 23]. The risk score was calculated by FRG expression (Exp i) and the corresponding coefficient (Coef i) as follows: risk score = ∑(Exp i × Coef i). FRGs were divided into high- and low-risk groups using the median of the risk scores as the cut-off value. Survival differences between high- and low-risk groups were shown by a Kaplan–Meier curve (K–M curve). The receiver operating characteristic (ROC) curve used R package “survivalROC” to evaluate the prediction ability of the signature by calculating the area under the curve (AUC). Then, the prognostic prediction ability of the FRG-based signature was verified in the testing and entire sets. Cox proportional hazard regression models were used in univariate and multivariate analyses of ChRCC patients to assess the prognostic value of the FRG-based signature and clinical variables. Covariates used in cox multivariable model included age, gender, and pathological stage. The association between the risk score and clinicopathological features was evaluated by the Wilcoxon rank sum test and shown in boxplots.

### Construction and validation of a nomogram combining the FRG signature and clinical features

A nomogram was constructed using the “rms” R package to predict the 5-year OS rate of ChRCC patients by FRG signature risk scores and clinical characteristics (age, gender, and pathological stage). A calibration curve was used to verify agreement between the actual and predicted OS assessed by this nomogram.

### Gene set enrichment analysis

Gene set enrichment analysis (GSEA) (version 4.2.3) was used to assess biology functions and pathways enriched in different groups using Gene Ontology (c5.go.v7.5.symbols.gmt) and the Kyoto Encyclopedia of Genes and Genomes (KEGG) (c2.cp.kegg.v7.5.symbols.gmt) gene set. Functions and pathways with a nominal *p*-value of < 0.05 and normalized enrichment score of > 1 were screened out.

### Immune cell infiltration and tumor microenvironment

The single sample GSEA (ssGSEA) algorithm was used to evaluate differences in immune cell infiltration and immune function scores between high- and low-risk groups through R package “GSVA” [24]. Immune, stromal, and ESTIMATE scores and tumor purity in the tumor microenvironment were obtained by the ESTIMATE algorithm. The expression levels of MHC and immune checkpoint genes were compared.

### Tumor mutational burden

The tumor mutation burden (TMB) in high- and low-risk groups was calculated by “maftools” R package. The Kaplan–Meier method was then used to compare the survival rates between the high and low mutation groups and the risk groups.

### Drug response analysis

The response of ChRCC patients in high- and low-risk groups to chemotherapeutic drugs in the Genomics of Drug Sensitivity in Cancer database was evaluated by the half maximal inhibitory concentration using “pRRophetic” R package. Additionally, we assessed the association between FRG expression and sensitivity to FDA-approved drugs by the CellMiner platform using Spearman approaches.

### Tissue samples and patients for external verification

Sixty ChRCC patients at the affiliated Hospital of Qingdao University from April 2008 to September 2020 were included in the study for external verification. We obtained paraffin-embedded tissues from the patients, including all tumor tissues and 32 para-cancer tissues, for immunohistochemical (IHC) staining. All patients had signed informed consent. Our study was approved by the Ethics Committee of the affiliated Hospital of Qingdao University and conformed to the Declaration of Helsinki.

### IHC

Paraffin-embedded tissue sections with a thickness of 4 μm were dewaxed with xylene and then rehydrated with gradient concentrations of ethanol. Antigen retrieval was performed in citrate buffer (pH 6.0; Zhongshan Biotechnology, Beijing, China) at 125 °C for 8 min in autoclave. Then, 3% hydrogen peroxide was used to block endogenous peroxidases. Anti-Transferrin Receptor (TFRC) (dilution: 1:400; ab214039, Abcam, Cambridge, UK) and anti-xCT (dilution: 1:600; ab37185, Abcam, Cambridge, UK) antibodies were applied to for 90 min at 4 °C. The sections were then washed and incubated with secondary antibodies for 20 min. Two-step diaminobenzidine staining was used for devilment, followed by hematoxylin counterstaining. Sections were scored semi-quantitatively by two pathologists who were blinded to the patient information in accordance with the staining intensity and area. The staining intensity was scored by the following criteria: 0 (negative), 1 (weak staining), 2 (moderate staining), and 3 (strong staining). The percentage of positive cells was stratified as follows: 0, < 5%; 1, 5–25%; 2, 26–50%; 3, 51–75%; 4, 76–100%. The IHC score was obtained by multiplying the two values.

### Statistical analysis

Statistical analysis was performed using R 4.1.3 software and GraphPad Prism version 8.0.1. We used the Wilcoxon rank-sum test and Mann-Whitney U test to compare the continuous variables and Spearman analysis to calculate correlation coefficients. Kaplan–Meier method was used to draw survival curve, and log-rank test was used to compare survival differences. *P* < 0.05 was considered to be statistically significant and was two-sided.

## Results

**Fig. 1:**
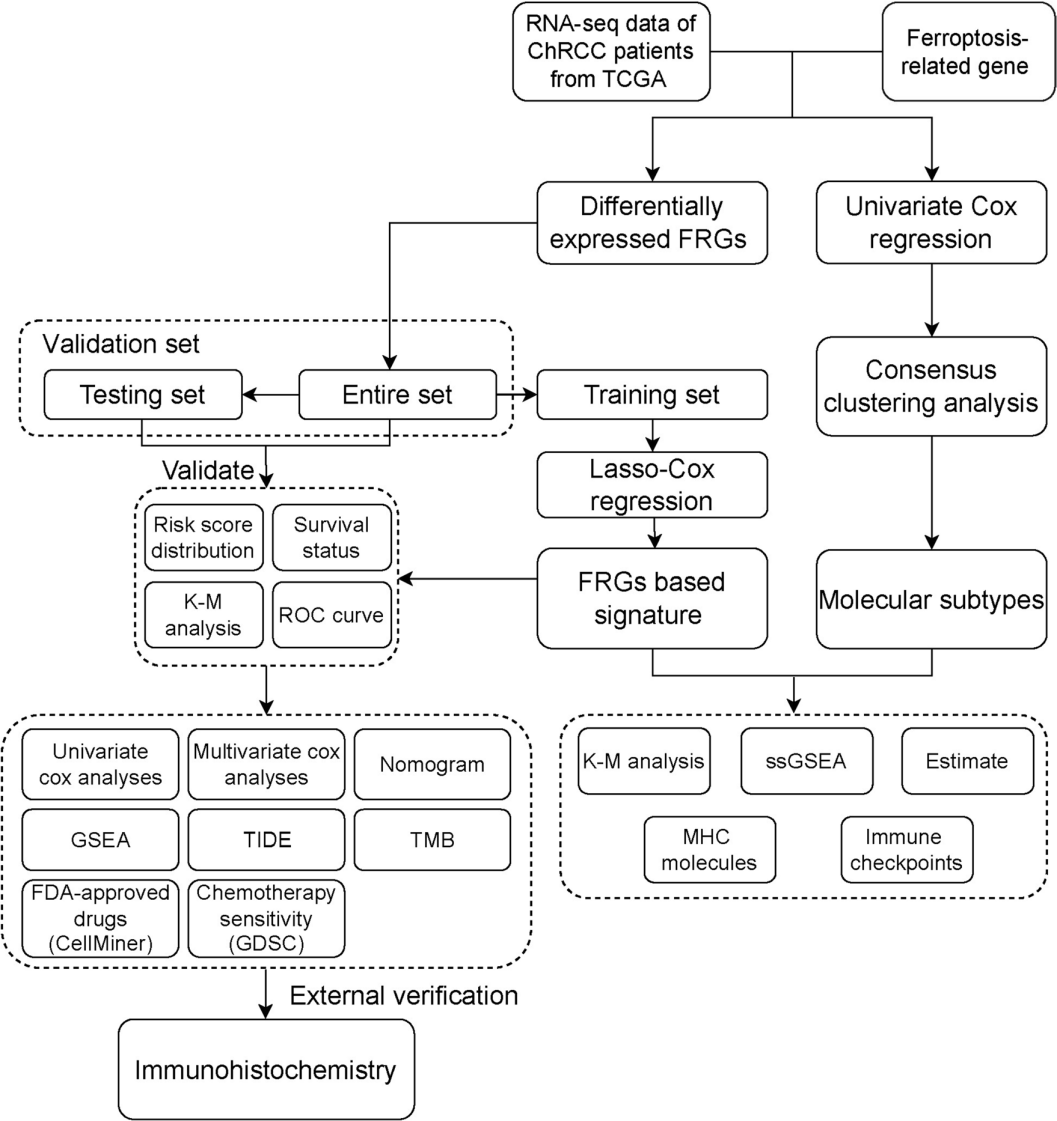
Flowchart of this study

A flowchart of this study is shown in Fig. 1. Sixty-six ChRCC patients from TCGA and 60 patients from our hospital were included in the study. Clinical characteristics are listed in Table 1.

**Table 1:** patients characteristics of TCGA and our hospital

Feature	TCGA (*N* = 66)	Our samples (*N* = 60)
Age (yrs)	50 (42–60)	50 (40–62)
Gender, n (%)		
Male	39 (60)	23 (38)
Female	26 (40)	37 (62)
Pathologic_stage, n (%)		
I	20 (31)	22 (37)
II	25 (39)	22 (37)
III	16 (25)	13 (22)
IV	4 (6)	3 (5)
Pathologic_T, n (%)		
pT1	20 (31)	22 (37)
pT2	25 (39)	22 (37)
pT3	18 (28)	14 (23)
pT4	2 (3)	2 (3)
Pathologic_N, n (%)		
pN0	39 (60)	58 (97)
pN1	5 (8)	2 (3)
Unknown	21 (32)	0 (0)
Pathologic_M, n (%)		
M0	34 (52)	58 (97)
M1	2 (3)	2 (3)
Unknown	29 (45)	0 (0)
Presence_of_sarcomatoid_features, n (%)		
No	62 (95)	58 (97)
Yes	3 (5)	2 (3)
Unknown	49 (43)	0 (0)

### Gene mutation and differential expression of FRGs

**Fig. 2:**
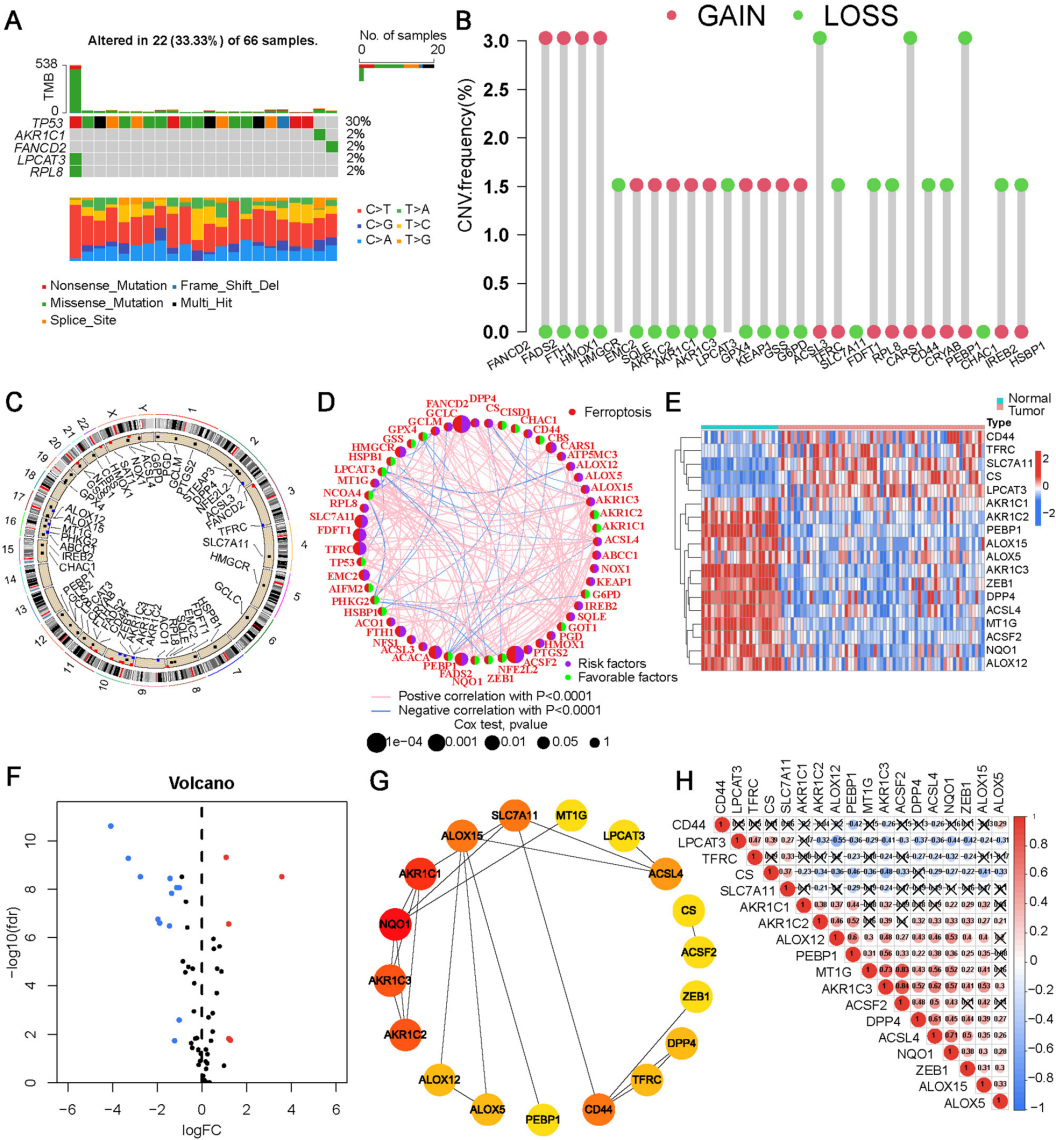
Genetic mutation and differential expression of FRGs in ChRCC. **A** Mutations frequencies of 60 FRGs in 66 ChRCC patients from TCGA. **B** Frequencies of CNV gain, loss, and non-CNV of FRGs. **C** Locations of CNV changes in FRGs on chromosomes. **D** Interaction network among FRGs in ChRCC. The thickness of the connection line indicates the strength of the interaction between genes. Heatmap (**E**) and volcano plots (**F**) for expression of differentially expressed FRGs in tumor and normal tissues. Red and blue represent upregulation and downregulation of genes, respectively. The PPI network (**G**) and correlation (**H**) between differentially expressed FRGs.

Sixty genes were selected as FRGs. The gene mutation patterns of FRGs in ChRCC are shown in Fig. 2A. Among the 66 samples, 22 samples (33.3%) had gene mutations, mainly in the TP53 gene (30% frequency). We analyzed the frequency of copy number variants (CNVs) in FRGs (Fig. 2B) and the locations of CNVs on chromosomes (Fig. 2C). The network showed a correlation between FRGs and the association between each FRG and prognosis (Fig. 2D). Eighteen FRGs were differentially expressed between cancer and para-cancer normal tissues (Table S2). The heatmap and volcano plot are shown in Fig. 2E, F. PPI network analysis and correlation coefficients of the differentially expressed FRGs are shown in Fig. 2G, H.

### Identification of molecular subtypes and corresponding tumor immune microenvironments

**Fig. 3:**
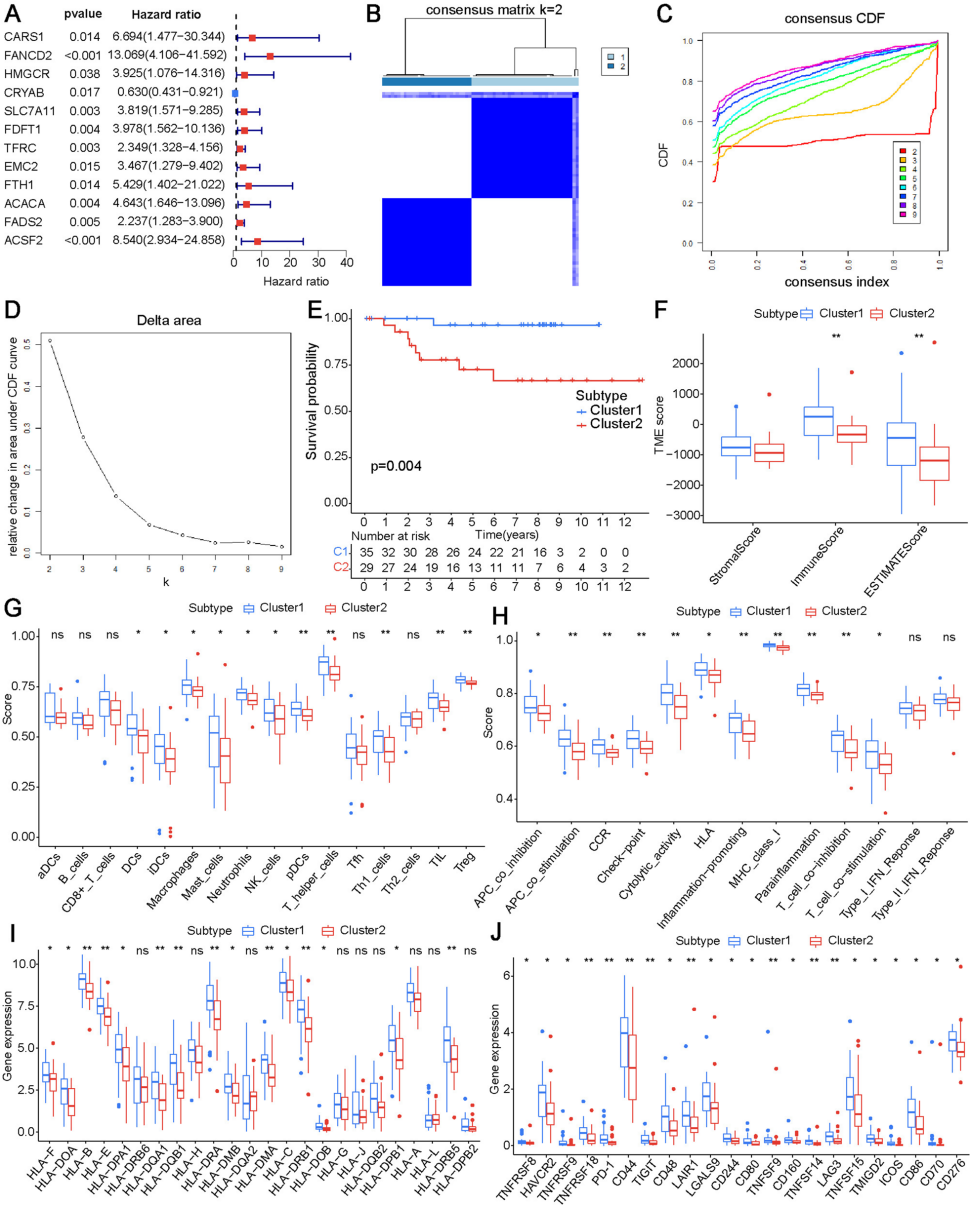
Identification of FRGs molecular subtypes and corresponding tumor immune microenvironments. **A** Forest plots showing *P*-values and HR (95% CI) of univariate cox regression analysis of FRGs and OS. **B** Consensus matrix heatmap defining two clusters (k = 2). **C** Curve of the consensus clustering cumulative distribution function. **D** Relative change in the area under CDF curve. **E** Survival curve of overall survival of clusters 1 and 2. **F** Stromal, immune, and ESTIMATE scores using the ESTIMATE algorithm. Immune cell infiltration (**G**) and immune function (**H**) score of the two clusters. Expression levels of MHC molecules (**I**) and immune checkpoint (**J**) genes in clusters

Twelve genes were identified as prognostic FRGs by univariate cox analysis (Fig. 3A). We performed consensus clustering analysis of these genes and found that the clustering effect was optimal when K = 2 (Fig. 3B–D). The K–M curve showed that cluster 2 had worse overall survival compared with cluster 1 (Fig. 3E). Next, we compared the immune microenvironments of clusters 1 and 2. In the cluster 1 subtype, the immune score and ESTIMATE score were higher in the ESTIMATE analysis (Fig. 3F). Cluster 1 had higher immune cells and immune-related function scores in the ssGSEA analysis (Fig. 3G, H). Because of the significant differences in immune infiltration between the two clusters, we assessed the association with the common immune checkpoints. The expression level of MHC and immune checkpoint genes were higher in the cluster 1 subtype (Fig. 3I, J).

### Construction of the FRG-based prognostic signature in ChRCC

**Fig. 4:**
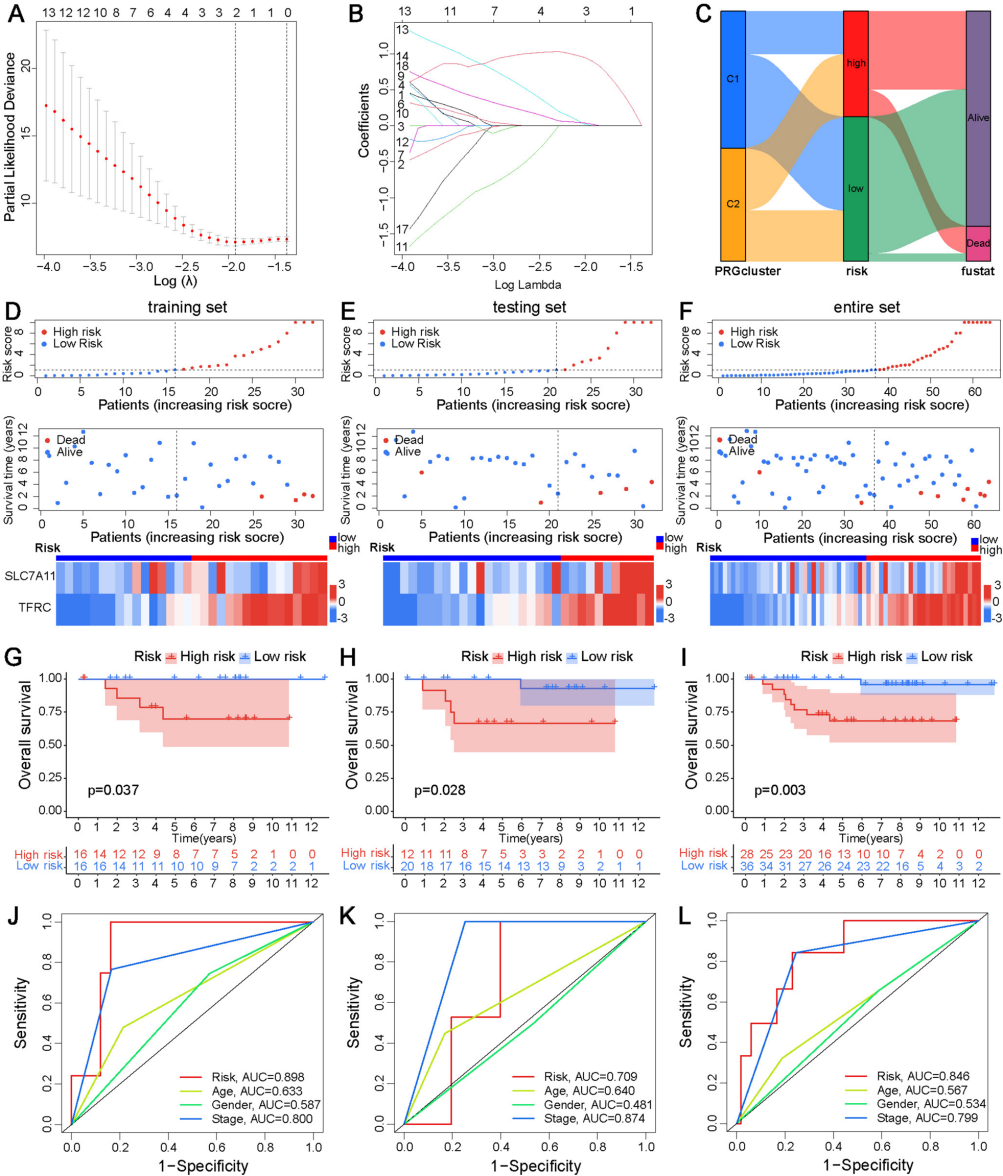
Construction and validation of the FRG-based prognostic signature. **A** LASSO regression analysis to select the optimal penalty lambda (λ). **B** LASSO coefficients of FRGs corresponding to log(λ). **C** Alluvial diagram revealing the distribution of patients in the two FRG subtypes and two risk groups. Scatter plots of the risk score, and survival status and time, and heatmap for expression of two genes in the training set (**D**), testing set (**E**), and entire (**F**) set. K–M curve of high- and low-risk groups in the training set (**G**), testing set (**H**), and entire set (**I**). ROC curves of the risk score and clinical characteristics in the training set (**J**), testing set (**K**), and entire set (**L**)

To further screen the genes included in the model, the prognostic signature was constructed by LASSO Cox regression analysis in the training set. When two genes (TFRC and SLC7A11) were included, the performance of the prognostic model was optimal (Fig. 4A, B). The risk score of ChRCC was calculated by the following formula: risk score = expression level of TFRC × 0.7940 + expression level of SLC7A11 × 1.9358. ChRCC patients were divided into high- and low-risk groups by the median risk score. An alluvial diagram revealed the patient distribution in the two FRG subtypes and two risk groups (Fig. 4C). The risk scores, corresponding survival status, and gene expression of high- and low-risk patients in the training set are displayed in Fig. 4D. Patients in the high-risk group had a worse overall survival rate than the low-risk group in Kaplan–Meier curves (Fig. 4G). The AUC of the FRG-based signature for 3-year OS was 0.898, which was higher than that of other clinical factors, indicating that this signature had a better predictive ability for survival (Fig. 4J).

### Validation of the FRG-based signature in the testing and entire sets

To determine the robustness of the model, the testing and entire sets used the same formula to calculate the risk score and were stratified by the median risk score of the training set. The distribution of FRG risk scores, survival status, gene expression, and Kaplan–Meier curves of testing and entire sets were consistent with the results of the training set (Fig. 4E, F). There was a significant association between higher risk scores and poorer overall survival in testing and entire sets (Fig. 4H, I). The AUC values for testing and entire sets were 0.709 and 0.846, respectively (Fig. 4K, L).

### Independent prognostic value of the FRG-based signature in TCGA for ChRCC

**Fig. 5:**
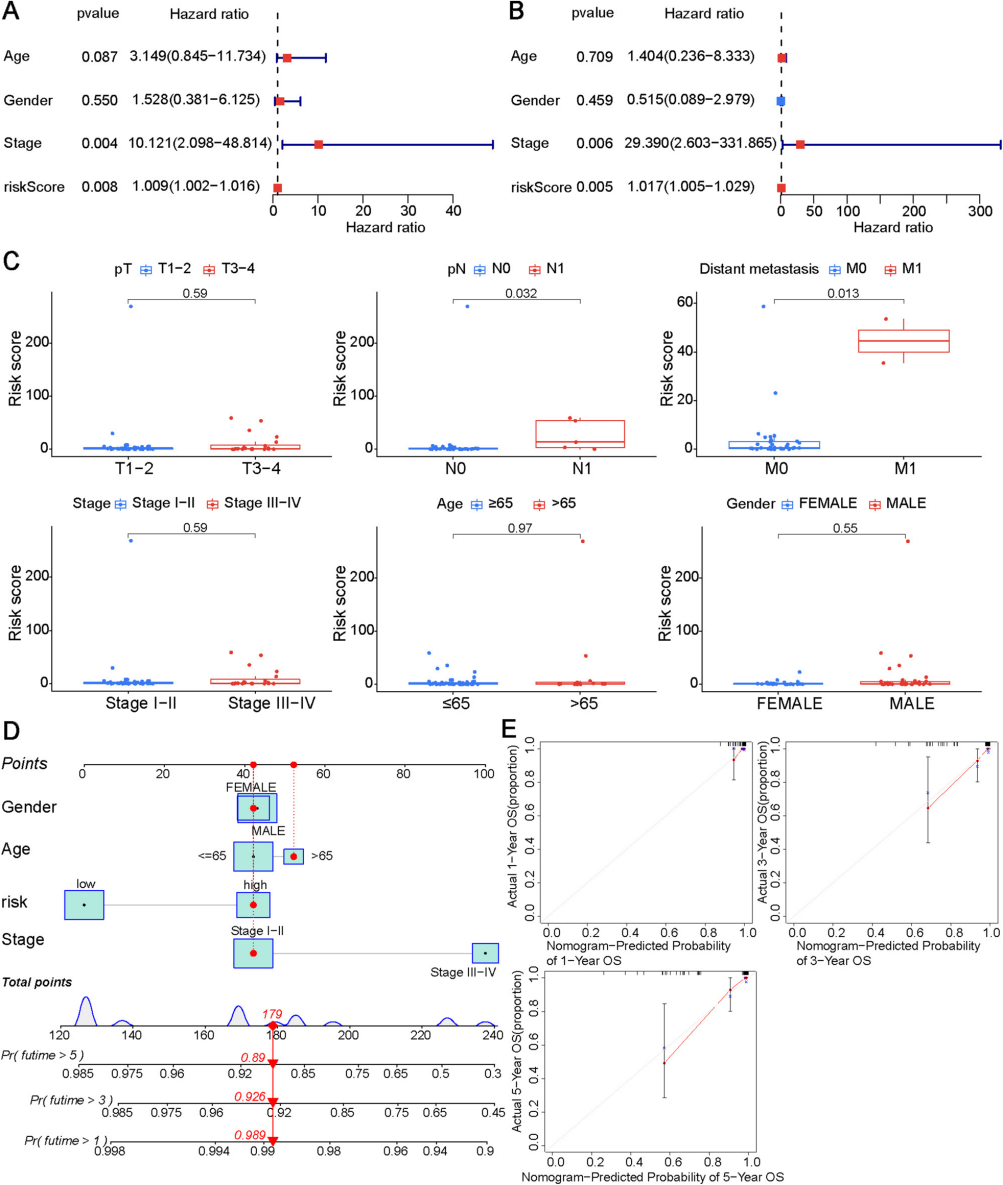
Cox proportional regression analysis and correlation with clinicopathological factors of the FRG-based signature. Forest plots of univariate (**A**) and multivariate (**B**) Cox proportional hazards regression analyses for overall survival of ChRCC patients in the entire set. **C** Association between the risk score, pathological stage, age, and gender. **D** Prognostic nomogram based on the FRG signature and clinical information. **E** Calibration curve of the nomogram to predict 1-, 3-, and 5-year OS.

To assess the independence of the FRG-based signature model for clinical application, we used univariate and multivariate Cox regression to analyze the clinical factors and the risk score. Univariate Cox regression analysis showed that the pathological stage (*P* = 0.004) and FRG risk score (*P* = 0.008) were significantly associated with overall survival (Fig. 5A). In multivariate analysis, the FRG risk score was an independent prognostic factor of overall survival (HR = 1.017, 95% CI = 1.005–1.029, *P* = 0.005; Fig. 5B). Through analysis of the correlation between the risk score and clinical information, we found that a higher risk score was significantly associated with regional lymph node metastasis (*P* = 0.032) and distant metastasis (*P* = 0.013), but not with an older age (*P* = 0.97), males (*P* = 0.6), advanced pathological stage III-IV (*P* = 0.6), and advanced pathological T stage (*P* = 0.6) (Fig. 5C).

### Construction and validation of a nomogram combining the FRG signature and clinical features

Subsequently, we constructed a nomogram model with clinical features (pathological stage, age, and gender). The nomogram used to predict 1-, 3-, and 5-year overall survival of ChRCC patients is shown in Fig. 5D. The calibration curve showed that the predicted values​​ from this nomogram were approximately in agreement with the actual values (Fig. 5E). The C-index of the nomogram was 0.877.

### Gene set enrichment analysis and tumor immune microenvironment

**Fig. 6:**
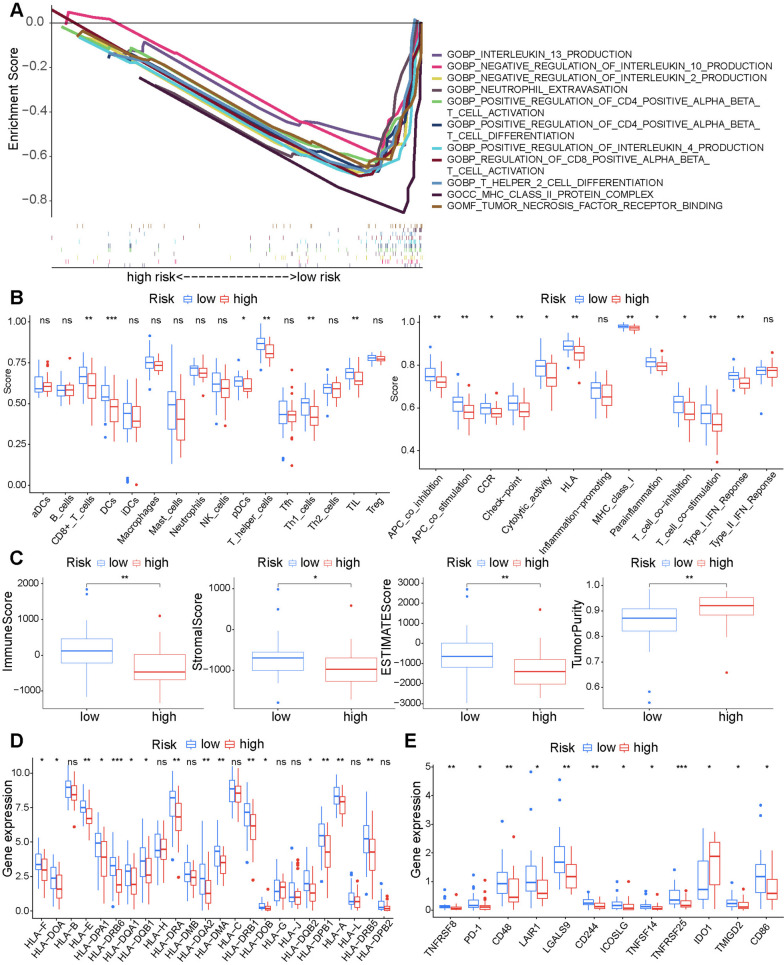
Immune landscape of the prognostic signature (**A**) Immune-related functions enriched in the low-risk group. Immune cells and immune-related functions (**B**), immune scores and stromal scores (**C**), expression levels of HLA (**D**) and common immune checkpoint genes (**E**) in the risk groups (***P < 0.001, **P < 0.01, *P < 0.05, ns, no significance)

To evaluate the pathways that may be involved in regulating tumourigenesis in the high- and low- risk group, GSEA was conducted. GSEA showed that many immune-related Gene Ontology biological processes were enriched in the low-risk group, including positive regulation of CD4-positive alpha beta T cell activation and differentiation, regulation of CD8-positive alpha beta T cell activation, T helper 2 cell differentiation, and tumor necrosis factor receptor binding (Fig. 6A). Therefore, we investigated the association between the FRG-based signature and tumor immune microenvironment. ssGSEA showed that the infiltration levels of immune cells (CD8^+^ T cell, DCs, T helper cells, Th1 cells, and TILs) and immune-related functions (APC co-inhibition, APC costimulation, cytokine–cytokine receptor, checkpoint, cytolytic activity, human leukocyte antigen, para-inflammation, T cell co-inhibition, T cell costimulation, and Type I IFN response) were higher in the low-risk group than in the high-risk group (Fig. 6B). The immune, stromal, and ESTIMATE scores were higher and tumor purity was lower in the low-risk group in accordance with the ESTIMATE algorithm (Fig. 6C). MHC molecules were significantly overexpressed in the low-risk group (Fig. 6D). Except for low expression of IDO1, the low-risk group was significantly associated with high expression of many immune checkpoint inhibitors (PD-1, CD30, LAIR1, CD48, LGALS9, CD244, ICOSLG, TNFSF14, TNFRSF25, TMIGD2, and CD86) (Fig. 6E).

### Comparison of the tumor mutation burden

**Fig. 7:**
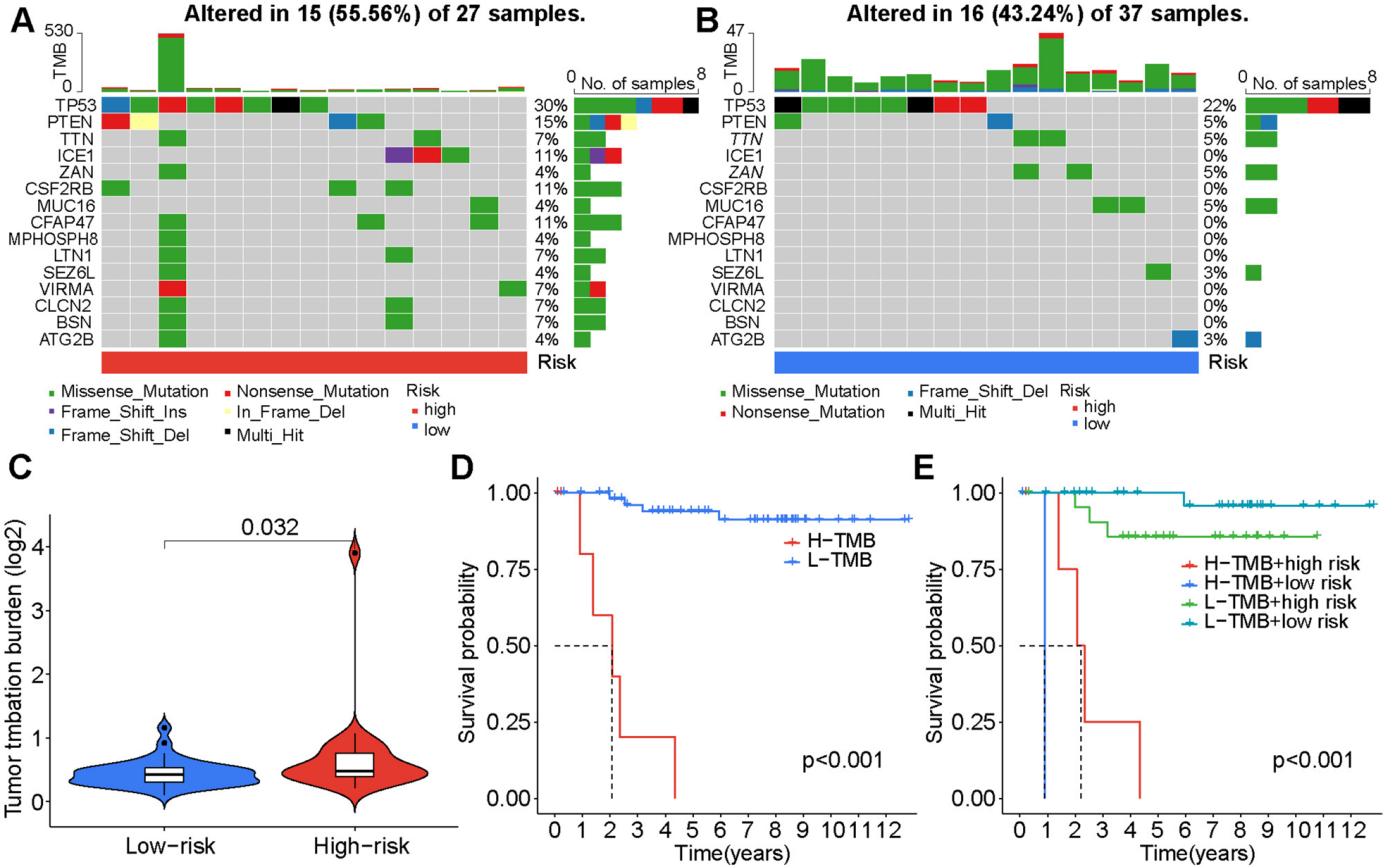
Waterfall map of the tumor mutation burden in the high-risk group (**A**) and low-risk group (**B**). **C** Violin plot of TMB differences between risk groups. **D** K–M curve of high and low TMB groups. **E** Survival curve of four combinations of TMB and risk scores

To assess the differences in genomic mutations between the high- and low-risk groups, we analyzed simple nucleotide variation data from TCGA. Genes with the highest mutation frequencies in the high-risk group were TP53 (30%), PTEN (15%), ICE1 (11%), CSF2RB (11%), and CFAP47 (11%) (Fig. 7A), and those in the low-risk group were TP53 (22%), PTEN (5%), ZAN (5%), and MUC16 (5%) (Fig. 7B). TMB in the high-risk group was significantly higher than that in the low-risk group (Fig. 7C). High TMB and high-risk groups had significantly worse overall survival than low TMB and low-risk groups (Fig. 7D, E).

### Drug response prediction

**Fig. 8:**
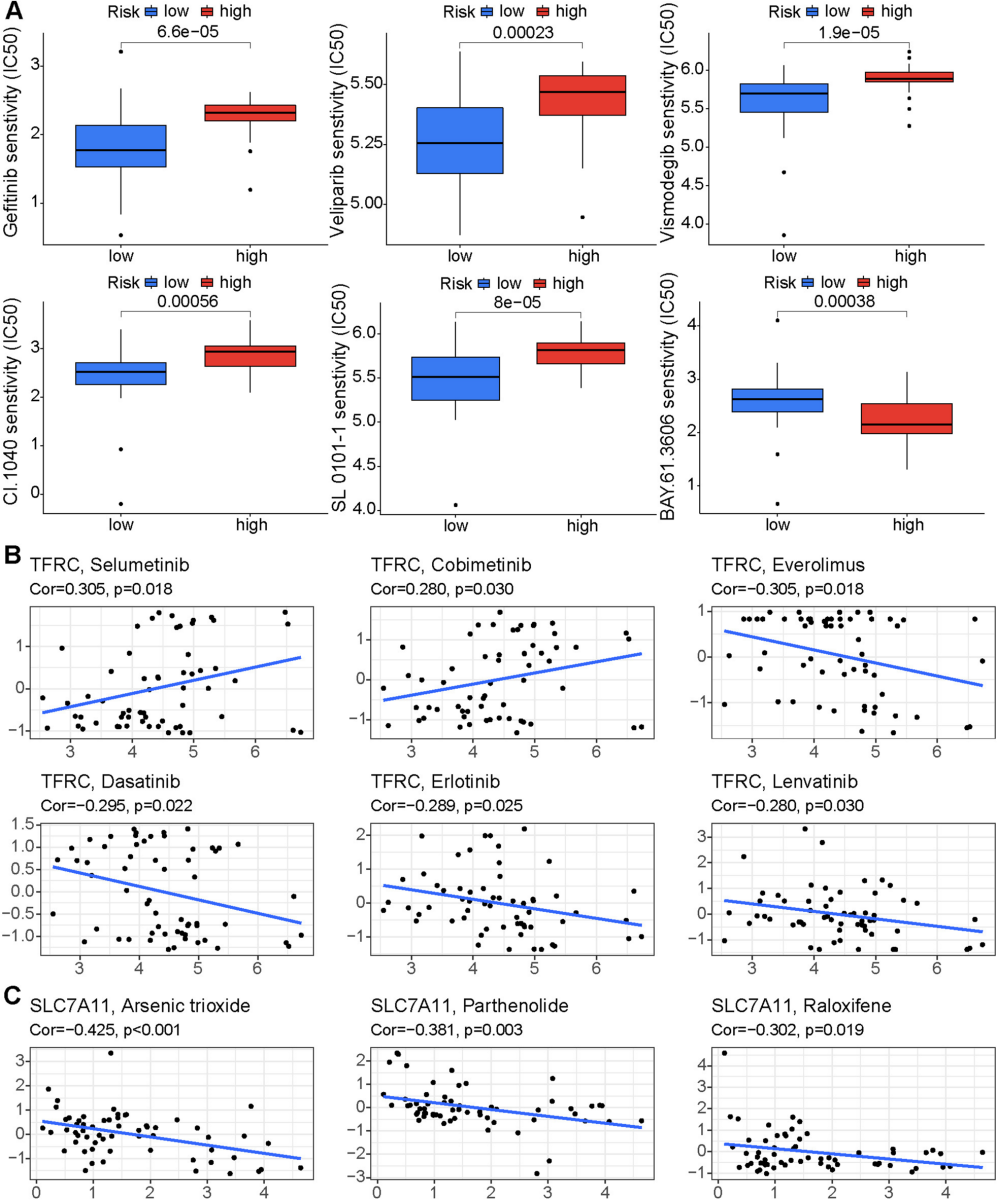
**A** Comparison of potential targeted drug sensitivities in high- and low-risk groups. Correlation between responses to some FDA-approved drugs and expression levels of TFRC (**B**) and SLC7A11 (**C**)

In the Genomics of Drug Sensitivity in Cancer database, we explored the association between the signature and targeted drugs, and found many drugs that were more sensitive in the high-risk group, including gefitinib, veliparib, vismodegib, PD184352, SL 0101-1, and BAY 61-3606 (Fig. 8A). Next, the correlation between the expression of genes involved in construction of the signature and the sensitivity of FDA-approved drugs was investigated using the CellMiner platform. Tumor cells with higher expression of TFRC had a better therapeutic effect when treated with selumetinib and cobimetinib, but had stronger resistance against everolimus, dasatinib, erlotinib, and lenvatinib (Fig. 8B). Tumor cells with higher expression of SLC7A11 were more resistant to arsenic trioxide, parthenolide, and raloxifene (Fig. 8C).

### Experimental verification of FRG expression levels

**Fig. 9:**
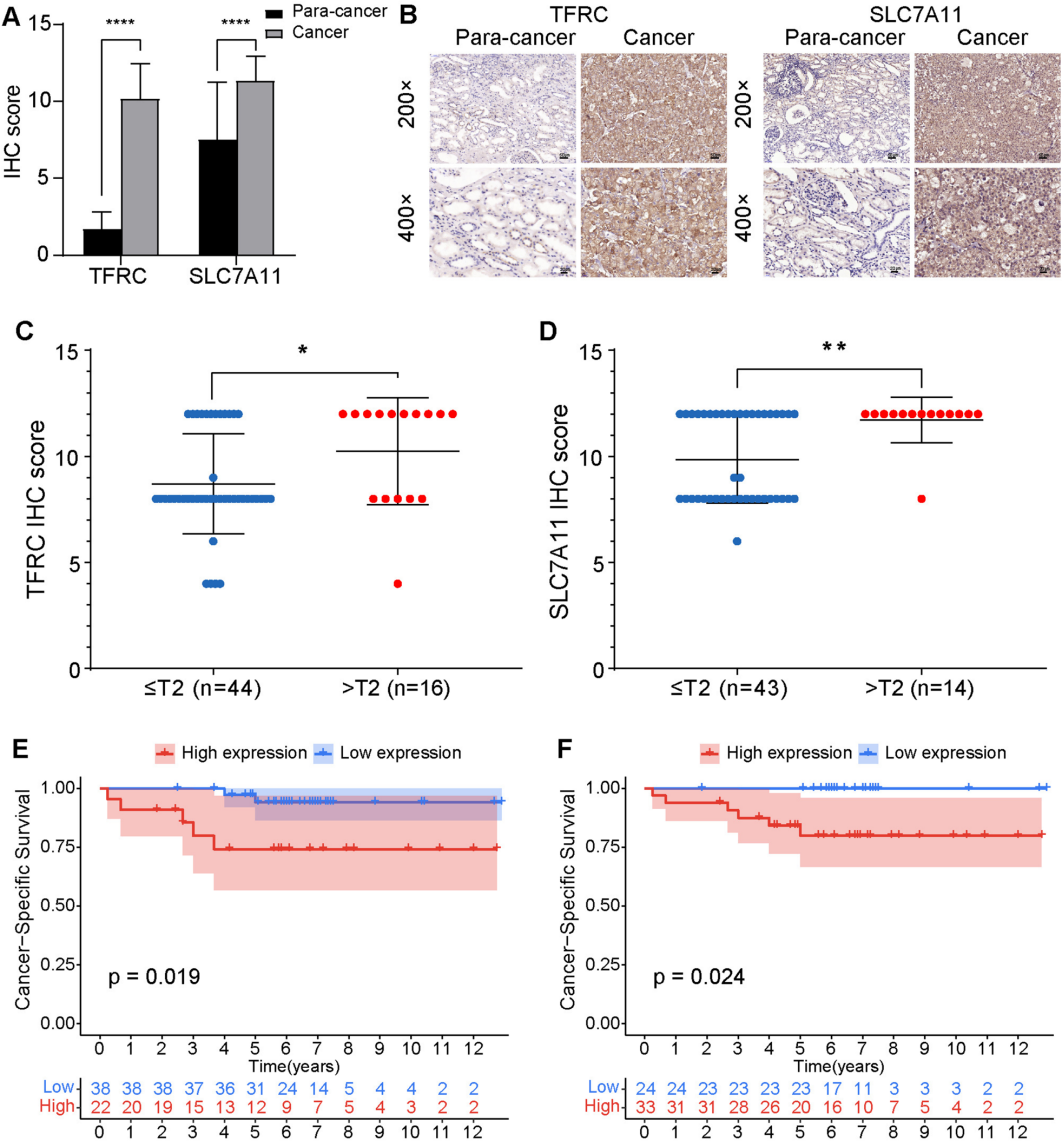
Experimental verification of FRGs in vitro. **A** Difference in immunohistochemical score of TFRC and SLC7A11 in cancer and para-cancer tissues. **B** Representative immunohistochemical staining of TFRC and SLC7A11 in cancer and para-cancer tissues. Comparison of IHC scores in different stages of TFRC (**C**) and SLC7A11 (**D**). Kaplan–Meier curves of high and low expression groups of TFRC (**E**) and SLC7A11 (**F**)

Boxplots of differential expression and K-M curves of FRGs (TFRC and SLC7A11) at the mRNA level in the TCGA-KICH cohort are shown in Supplementary Figure S1A–D. Furthermore, to verify the accuracy of the FRG signature, we investigated the expression of the signature genes (TFRC and SLC7A11) in clinical samples from ChRCC patients by IHC analyses. By immunohistochemical staining of 32 pairs of tumor and para-cancer tissues from our hospital, we found that the expression levels of TFRC (*P* < 0.0001) and SLC7A11 (*P* < 0.0001) in tumor tissues were significantly higher than those in para-cancer tissues (Fig. 9A). Representative IHC-stained sections are shown in Fig. 9B. Immunohistochemical analysis of tumor tissues from 60 ChRCC patients showed a significant association between higher expression of TFRC (*P* = 0.026; Fig. 9C) and SLC7A11 (*P* = 0.004; Fig. 9D) and a more advanced stage. K–M curves showed that high expression of TFRC and SLC7A11 was remarkably associated with worse survival (log-rank: TFRC, *P* = 0.019; SLC7A11, *P* = 0.024) (Fig. 9E, F).

## Discussion

The link between iron and tumors is a hot research topic. Numerous studies have found that excess iron in the body increases the incidence of cancer, and iron is associated with tumor progression [25]. Ferroptosis, an iron-dependent form of cell death, plays an important role in tumorigenesis, tumor microenvironment, and immunotherapy [17]. FRGs are overexpressed in RCCs and associated with an advanced stage and worse prognosis, mainly in clear cell renal cell carcinoma (ccRCC) and papillary renal cell carcinoma (pRCC) [26–29]. The prognostic value of FRGs in chromophobe renal cell carcinoma is unclear. Therefore, we evaluated its association with the immune microenvironment.

The consensus cluster analysis of ChRCC on the basis of FRGs was carried out to identify two clusters, which established molecular subtypes of ChRCC. Cluster 1 with more immune cell infiltration corresponded to the immune-inflamed phenotype, which was consistent with higher expression of MHC and immune checkpoint molecules and a better prognosis [30]. Cluster 2 lacking immune infiltration was closer to the immune desert phenotype.

TFRC and SLC7A11 genes were used to construct the FRG-based signature, which had the best predictive efficiency. The TFRC gene encodes the transferrin receptor, a membrane glycoprotein that binds to transferrin and transfers iron into cells. High expression of TFRC has been found in many tumors, such as lung, prostate, bladder, thyroid, and cervical cancers, and its overexpression is associated with a worse prognosis [31–36]. High expression of TFRC promotes DSS-induced colonic epithelial cell death by activating the IL-6/IL-11-Stat3 pathway, which leads to mucosal injury and the occurrence of colon cancer [37]. Senyilmaz et al. reported that TFRC activates the JNK signaling pathway to regulate mitochondrial functions and induces tumor cell proliferation [38]. Additionally, TFRC enhances cellular production of ROS and mitochondrial respiration, leading to the development of pancreatic ductal adenocarcinoma and induction of c-Myc lymphoma-mediated tumorigenesis [39, 40]. SLC7A11 (also called xCT) is a transmembrane protein that is imports cystine into cells for glutathione intracellular synthesis [41]. This process inhibits ferroptosis by enhancing detoxification of lipid peroxidation regulated by GPX4. SLC7A11 is a prognostic risk factor for many pathological types of RCC [42] and has been used to establish a prognostic model for ccRCC [16, 43, 44]. High expression of SLC7A11 is significantly associated with poor differentiation and a more advanced stage of hepatocellular carcinoma and is an independent prognostic factor for survival [45, 46]. SLC7A11 also promotes the growth and development of non-small cell lung cancer and corresponds to shorter 5-year survival of patients [47]. The mechanism of SLC7A11 promoting tumor growth has been widely studied. p53 and BAP1 (tumor suppressors) mutations [14, 48], increased expression of OTUB1 (tumor growth-promoting protein) [49], and loss of KRAS (proto-oncogene) [50] are associated with SLC7A11 overexpression, causing inhibition of ferroptosis and promotion of tumor growth.

Some studies have found that ferroptosis is associated with tumor immunity of ccRCC and has the potential to become a novel target for immunotherapy [16, 29]. We studied the association between the FRG-based signature and tumor microenvironment of ChRCC. As a major anti-tumor effector cell, high levels CD8^+^ T cells are associated with a better prognosis of ChRCC patients [51], which is consistent with our findings. Low-risk subgroups with higher levels of CD8^+^ T cells had a better prognosis, which also confirmed the difference in the immune microenvironment between ChRCC and ccRCC, and higher infiltration of CD8^+^ T cells has been associated with shorter survival of ccRCC patients [52]. Higher levels of dendritic cells, T helper cells, CD4^+^ T cells, and tumor-infiltrating lymphocytes were also observed in the low-risk group. PD-L1 on dendritic cells is the direct-action point of immune checkpoint inhibitors and determines the effect of immunotherapy. Tumor immunotherapy promotes transmission of signals on dendritic cells from CD4^+^ T cells to CD8^+^ T cells, and finally enhances the activity of cytotoxic T lymphocytes. By analysis of ssGSEA and ESTIMATE algorithm, we found that the level of immune cell infiltration was higher and immune-related functions were more active in the low-risk group than in the high-risk group, which indicates that the low-risk group had better tumor immunity and better sensitivity to immunotherapy. The low-risk group had higher expression levels of MHC and immune checkpoint-related genes. Gu et al. found that deletion of MHC molecules is the main reason for immunotherapy resistance [53]. These results suggest that the high-risk group had a higher risk of tumor immune evasion [54–56].

TMB indicates the number of mutations that generate neoantigens presented to T cells by major histocompatibility complexes. A higher TMB represents an easier recognition opportunity for T cells because of the presence of more neoantigens, which is associated with the effect of immunotherapy [54]. In our study, a higher TMB was observed in the high-risk group and was associated with a poorer prognosis. A high TMB may be due to a higher degree of malignancy or tumor progression in high-risk groups, and thus, immunotherapy may not be less effective in the low-risk group [54].

Immunotherapy has become the standard treatment for renal cell carcinoma, but clinical trials showed that its therapeutic effect on ChRCC was limited. Our study suggested screening for sensitive types of ChRCC for systemic therapy. Combination therapy is becoming increasingly important in clinical applications because the effect of single immunotherapy appears to be poor. The high-risk group was more sensitive to gefitinib, veliparib, and vismodegib, suggesting that the high-risk group may benefit from targeted therapy. Higher expression of TFRC is associated with a better response to selumetinib and cobimetinib, and poorer sensitivity of tumor cells to everolimus, dasatinib, erlotinib, and lenvatinib. Selumetinib, a selective MEK1 inhibitor, increases the killing effect of everolimus in RCC [57], and the combination of MEK inhibitor cobimetinib and cabozantinib reduces drug resistance of RCC [58]. Everolimus, a mTOR inhibitor, prolongs the survival of ChRCC patients, and dasatinib and erlotinib are potential therapeutic drugs for ChRCC [59]. Hutson et al. found that the objective response rate of lenvatinib combined with everolimus for ChRCC treatment was up to 44% [60]. Lower expression of SLC7A11 is associated with higher responses of cancer cells to arsenic trioxide, parthenolide, and raloxifene that inhibit the proliferation or migration of RCC cells and are a potential therapeutic strategy for RCC [61–63].

There are some limitations in our study. First, RNA sequences of ChRCC can only be obtained from TCGA database, and other databases such as GEO have a small sample size and no clinical information. Second, the number of patients with comprehensive therapy such as immunotherapy for ChRCC at our center was too small to verify the predictive effect of the risk signature on the response to immunotherapy or targeted therapy.

## Conclusions

In summary, molecular subtypes and prognostic signature based on FRGs in chromophobe renal cell carcinoma were identified and significantly associated with the tumor microenvironment, tumor mutation burden, immunotherapy and targeted therapy response, which may help clinicians judge the prognosis of patients and formulate a comprehensive treatment plan.

### Supplementary Information

**Additional file 1: Table S1. **Sixty ferroptosis-related genes.** Table S2. **Eighteen FRGs differentially expressed between cancer and para-cancer normal tissues.** Figure S1.** The expression levels of TFRC (A) and SLC7A11 (B) for ChRCC between cancer and para-cancer tissues in the TCGA database. Survival curve of high and low expression groups of TFRC (C) and SLC7A11 (D) for the patients with ChRCC in the TCGA.

## Data Availability

The datasets analyzed in the current study are available in TCGA repository, [https://portal.gdc.cancer.gov/repository?facetTab=files]. The original contributions presented in the study are included in the article/Supplementary Material. Further inquiries can be directed to the corresponding authors.

## Abbreviations

TCGA: The cancer genome atlas
ChRCC: Chromophobe renal cell carcinoma
FRGs: Ferroptosis-related genes
KM: Kaplan–Meier
ROC: Receiver operating characteristic
C-index: Concordance index
AUC: Area under the receiver operating characteristic curve
LASSO: Least absolute shrinkage and selection operator
GSEA: Gene set enrichment analysis
ssGSEA: Single-sample gene set enrichment analysis
TFRC: Transferrin Receptor
SLC7A11: Solute Carrier Family 7 Member 11
