# Supplementary material for: Identification and validation of a ferroptosis-related signature for prediction of the prognosis and tumor microenvironment in patients with chromophobe renal cell carcinoma
Source: BMC Cancer. 2023 Nov 8;23:1079. doi: 10.1186/s12885-023-11589-5 (PMC10634106; doi:10.1186/s12885-023-11589-5)
Supplement: Supplementary file 1 — Additional file 1: Table S1. Sixty ferroptosis-related genes. Table S2. Eighteen FRGs differentially expressed between cancer and para-cancer normal tissues. Figure S1. The expression levels of TFRC (A) and SLC7A11 (B) for ChRCC between cancer and para-cancer tissues in the TCGA database. Survival curve of high and low expression groups of TFRC (C) and SLC7A11 (D) for the patients with ChRCC in the TCGA. [file 12885_2023_11589_MOESM1_ESM.docx]

| **Table S1.** Sixty ferroptosis-related genes | | |
| --- | --- | --- |
| ACSL4 | GSS | STEAP3 |
| AKR1C1 | HMGCR | NFS1 |
| AKR1C2 | HSPB1 | ACSL3 |
| AKR1C3 | CRYAB | ACACA |
| ALOX15 | LPCAT3 | PEBP1 |
| ALOX5 | MT1G | ZEB1 |
| ALOX12 | NCOA4 | SQLE |
| ATP5MC3 | PTGS2 | FADS2 |
| CARS1 | RPL8 | NFE2L2 |
| CBS | SAT1 | KEAP1 |
| CD44 | SLC7A11 | NQO1 |
| CHAC1 | FDFT1 | NOX1 |
| CISD1 | TFRC | ABCC1 |
| CS | TP53 | SLC1A5 |
| DPP4 | EMC2 | GOT1 |
| FANCD2 | AIFM2 | G6PD |
| GCLC | PHKG2 | PGD |
| GCLM | HSBP1 | IREB2 |
| GLS2 | ACO1 | HMOX1 |
| GPX4 | FTH1 | ACSF2 |

| **Table S2.** Eighteen FRGs differentially expressed between cancer and para-cancer normal tissues |
| --- |
| ACSL4 |
| AKR1C1 |
| AKR1C2 |
| AKR1C3 |
| ALOX15 |
| ALOX5 |
| ALOX12 |
| CD44 |
| CS |
| DPP4 |
| LPCAT3 |
| MT1G |
| SLC7A11 |
| TFRC |
| PEBP1 |
| ZEB1 |
| NQO1 |
| ACSF2 |


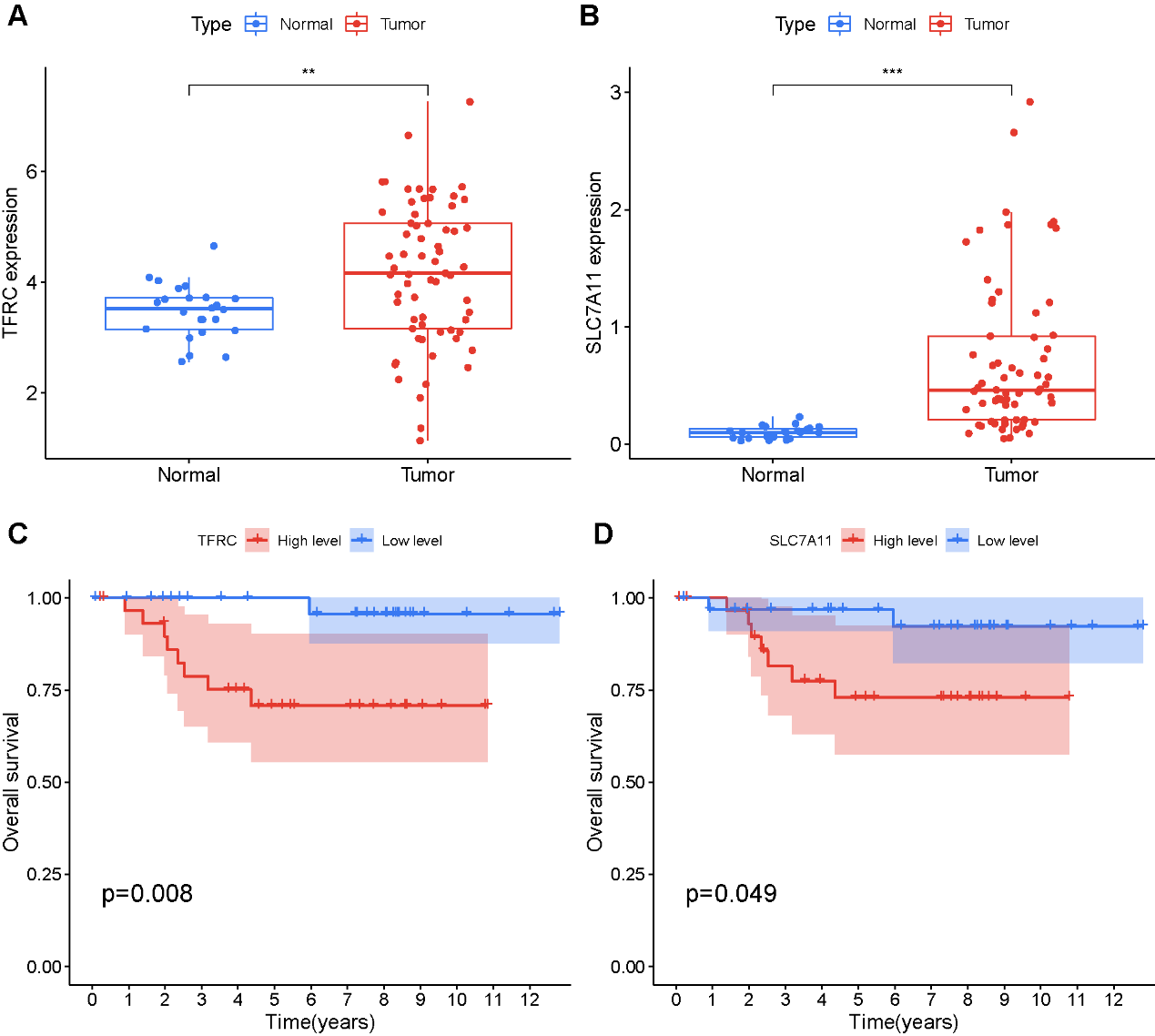


**Figure S1.** The expression levels of TFRC **(A)** and SLC7A11 **(B)** for ChRCC between cancer and para-cancer tissues in the TCGA database. Survival curve of high and low expression groups of TFRC **(C)** and SLC7A11 **(D)** for the patients with ChRCC in the TCGA.
